# Spatiotemporal localization of sex difference in plantar pressure distribution during self paced walking in healthy Japanese adults

**DOI:** 10.1371/journal.pone.0349134

**Published:** 2026-05-15

**Authors:** Hirokazu Doi, Natsumi Furuta, Hyunchul Yoon, Kazuo Funato

**Affiliations:** 1 Department of Information and Management Systems Engineering, Nagaoka University of Technology, Nagaoka, Japan; 2 Graduate School of Sport System, Kokushikan University, Setagaya, Japan; UC: University of Calgary, CANADA

## Abstract

**Objectives:**

There is notable sex difference in the manner of locomotion, but researchers are yet to obtain the comprehensive information about the biomechanical difference in gait pattern between males and females. The present study aimed to investigate sex differences in plantar pressure distribution during self-paced walking using a cluster-based permutation approach.

**Method:**

Plantar pressure data were collected from 24 healthy males and 68 healthy females using a pressure-sensor footplate. Group comparisons were conducted for basic gait parameters as well as pressure-related metrics, including maximum force, peak pressure, and contact area size. Additionally, time-series characteristics of total force were compared between sexes. A cluster-based permutation test was used to identify spatial and temporal regions with significant sex differences in plantar pressure distribution at high resolution.

**Results:**

Analyses revealed that females exhibited shorter step durations and higher cadence compared to males, attributable in part to differences in the duration of the late stance phase. Females also demonstrated higher weight-normalized plantar pressures across most of the stance phase. Sex-specific differences in plantar pressure distribution were localized to the calcaneal and second metatarsal regions.

**Conclusion:**

Spatially localized pattern of sex difference in the plantar pressure indicates that biomechanical factors may contribute to the sex difference in the incidence rates of clinical conditions such as stress fracture. There was also a hitherto unreported pattern of sex difference in the time-series of total force, that may be related to sex-specific strategy to keep a self-preferred walking speed.

## 1. Introduction

Locomotor movement is generated by a series of coordinated, time-structured activations of the musculoskeletal system orchestrated by the central pattern generator [1]. Gait represents a stereotyped movement, characterized by its complexity in both style and the relative timing of its constituent motion components [2,3]. It is well recognized that notable sex differences exist in gait, as illustrated by the classic study by [4], which demonstrated that observers could reliably discern the sex of a walker based solely on an animation of joint motion. Given that such sex differences in gait are perceptible even to the human eye [5], a detailed characterization of these differences may provide insights into the etiology of clinical conditions with sex-dependent incidence rates, such as osteoarthritis [6] and stress fractures [7]. Despite the apparent importance of this issue, researchers have yet to obtain a comprehensive understanding of sex differences in locomotor movement [8,9].

Previous studies have identified sex differences in basic gait parameters, with females generally exhibiting shorter step lengths [8,10], that tend to be compensated for by higher cadence [10], compared to males. Detailed kinematic analyses have reported greater pelvic obliquity range [8,11,12] and a wider range of ankle motion [8,13–15] in females. Nevertheless, aside from these replicable observations, the existing findings on sex differences in locomotor movement are often fragmented or inconsistent across studies [15].

When a foot makes contact with the ground, force is exerted to the ground surface, and the ground pushes back the foot with the equal force, generally termed “ground reaction force (GRF)”. Plantar pressure, as measured in “pedobarometry”, is the distribution of the vertical component of GRF, and provides ample information on the foot kinetics during stance and motion [16]. As such, plantar pressure measurement has been widely utilized in various domains of gait research, including the assessment of gait in patients with neurological and psychiatric conditions [17,18] and the investigation of motor development during early infancy [19,20].

Although limited in number, several previous studies have compared plantar pressure distributions between adult males and females. Many studies found larger contact area in males than in females [21,22]. Several studies compared the peak pressure and maximum force across stance phase between males and females. Murphy et al [23] found no sex difference in the normalized plantar pressure in the midfoot regions. Later studies found sex difference in the parameters reflecting the plantar pressure distribution, but the reported pattern of sex difference is not convergent across studies. Putti et al [21] reported higher maximum force in the heel and the metatarsal regions in males with no sex difference in peak pressure. Chung et al [22] reported generally higher peak pressure and maximum force in males than females. In contrast, Chinese females show higher plantar pressure in the medial forefoot including hallux and 2^nd^-5^th^ toes than males [24]. Similarly, early adolescent females are reported to show larger peak pressure and maximum force than the age-matched males [25].

Several limitations in the previous studies on sex difference in the plantar pressure deserve attention. First, it is a common practice in plantar pressure research to segment the contact area into discrete foot regions [21,24,25], and to compute plantar pressure metrices such as peak pressure within each segmented area. However, since the pressure value is averaged across multiple sensors within segmented region, this analytic procedure fails to capture the fine-grained spatial information as to the location of sex difference in plantar pressure distribution [19]. Moreover, there is some arbitrariness in the way the foot region is segmented. Indeed, [21] argued the possibility that researchers might miss subtle sex differences in plantar pressure distribution when adopting spatially-coarse segmentation.

Second, relatively little attention has been paid to the sex difference in the time-series of dynamic change of plantar pressure distribution. Parameters reflective of plantar pressure distribution typically exhibit distinct temporal patterns. For example, the time-series of total force shows a typical tri-phasic or “M-shaped” pattern [26,27]. However, sex-related variations in these time-series patterns throughout the gait cycle remain poorly understood. Similarly, computation of the center of pressure (COP) is a practical and reliable way to capture the spatiotemporal dynamics of the plantar pressure distribution [28]. Chiu, Wu and Chang [29] reported that the COP moved more medially in males than in females during stance phase based on the plantar pressure data [29]. However, systematic investigation into the sex difference in COP progression is limited.

The present study aims to elucidate sex differences in plantar pressure distribution during self-paced walking in a comprehensive way by filling in the gaps in knowledge described above. For this purpose, novel analytic approaches were adopted in the present study, in addition to the analysis of the sex differences in the oft-reported metrices of plantar pressure, including maximum force, peak pressure and contact area size.

To avoid the problems associated with the method of foot region segmentation [19], the location of sex difference in plantar pressure distribution was spatially localized with high spatial resolution at load response, mid-stance and pre-swing sub-phases of stance phase. More specifically, the spatial clusters, *i.e.,* foot regions, with significant sex difference were detected by cluster-based permutation test [30,31]. Cluster-based permutation test is a statistical method to detect spatial or temporal cluster with statistically significant effect of interest. Since first introduced as a non-parametric alternative to statistical parametric mapping [32] to analyze 3D neuroimaging data [30], it is widely used in the statistical comparison of medical images and time-series [31,33]. The first novelty of the present study lies in the adaptation of this statistical method to map sex differences in plantar pressure distribution at high spatial resolution without arbitrary segmentation of foot regions.

It is widely acknowledged that females have a higher incidence rate of stress fractures than males [7,34]. Previous research has confirmed an association between stress fractures and plantar pressure, particularly in the metatarsal regions, during athletic movements including running [35]. However, little is known about the association between stress fractures and the repetitive application of GRF to the foot during everyday walking. Considering the higher incidence rate of stress fractures in females [7,34], together with the established link between bone stress injury and plantar pressure [35], we hypothesized that females exhibit higher plantar pressure normalized to body weight in foot regions susceptible to stress fractures.

Relatively little attention has been paid to sex differences in the temporal progression of plantar pressure throughout the gait cycle, compared to sex difference in its spatial distribution. The second key novelty of the present study is the application of cluster-permutation statistics to the temporal courses of plantar pressure parameters and COP trajectories, an approach not reported in the previous studies, thereby enabling precise identification of the temporal windows during which sex differences in gait kinetics are observed.

With regard to the temporal dynamics of plantar pressure distribution, the present study analyzed the sex difference in the length of the temporal interval between neighboring peaks in the “M-shaped” time-series of total force. We further localized the temporal window during which sex difference is detected in the time-series of the pressure metrics, *i.e.,* total force, averaged pressure and contact area size, by the cluster-based permutation test. The sex difference in the spatiotemporal dynamics of plantar pressure distribution was explored further by localizing the temporal window with significant sex difference in the COP trajectory.

## 2. Method

### 2.1. Participants

A total of 24 healthy males and 68 females participated in the present study after providing written informed consent. The participants’ age, height, weight, and BMI were summarized separately for males and females in Table 1. The study protocol was reviewed and approved by the ethical committee of the faculty of Graduate School of Sport System, Kokushikan University (Approval No. 21014) in accordance with the Declaration of Helsinki.

**Table 1:** Age and the anthropometric measures of the participants. In the parentheses are the standard deviations.

	Female	CI Lower	CI Upper	Male	CI Lower	CI Upper	*t*	*p*-value	Cohen’s *d*
N	68			24					
**Age (yrs)**	34.5	30.87	38.13	33.7	27.52	39.9	0.23	0.822	0.05
	(14.9)			(14.4)					
**Height (cm)**	159.4	158.24	160.60	172.8	170.65	174.86	−11.33	<.001**	−2.72
	(4.8)			(4.9)					
**Weight (kg)**	54.9	53.12	56.69	70.1	66.22	74.07	−7.27	<.001**	−1.93
	(7.3)			(9.1)					
**BMI**	21.6	20.95	22.23	23.5	22.27	24.70	−2.82	0.008**	−0.7
	(2.6)			(2.8)					

**p < .01, uncorrected for multiple comparisons, CI Lower: Lower limit of the 95% confidence interval, CI Upper: Upper limit of the 95% confidence interval.

### 2.2. Apparatus

Plantar pressure data were collected using the Novel emed XL system (1529 × 504 mm, Novel GmbH®). The footplate consists of 25,344 pressure sensors, each measuring 5 mm × 5 mm, arranged over a 440 mm × 1440 mm active measurement area. Each sensor recorded pressure with a resolution of 5 kPa (0.5 N/cm²) and a detection threshold of 10 kPa (1 N/cm²). The sampling frequency was set at 100 Hz.

### 2.3. Procedure

Plantar pressure data were collected using the midgait procedure, the reliability of which for plantar pressure measurement is well established [36]. The walkway was approximately 15 m in length. The foot plate was placed at the midpoint of the walkway, approximately 7 m from the starting point. Participants were instructed to walk barefoot across the walkway at a self-selected pace while maintaining their gaze straight ahead. The stability of walking speed was monitored online through visual inspection by the experimenters. A trial was considered valid when pressure data for two consecutive steps were successfully recorded. Trials in which participants looked downward while walking were excluded. Each participant repeated the task until one valid trial was obtained.

### 2.4. Analysis

#### 2.4.1. Preprocessing.

For subsequent analysis, individual contact areas were segmented from the raw pressure data. Based on the visualized images of the plantar pressure, the frames and spatial regions corresponding to each stance phase were identified. The onset of a stance phase was defined as the first frame in which any sensor recorded a non-zero pressure value within the contact area, while the offset was defined as the last frame in which non-zero pressure was detected in any sensor.

#### 2.4.2. Parameter extraction.

2.4.2.1 Basic Gait Parameters. Step length, step duration, cadence, walking speed, and foot angle were extracted as basic gait parameters from the plantar pressure distribution data. Gait parameters were computed based on the first two available steps.

Step length (m) was defined as the distance between the heel contact position of one foot and the next contact position of the opposite foot along the direction of progression. Step duration (sec) was defined as the temporal interval between the consecutive heel contacts of the opposite feet. Cadence (steps/min) was computed by dividing 60 sec by the step duration in seconds. Walking speed (m/sec) was calculated by dividing step length by step duration.

**Fig 1:**
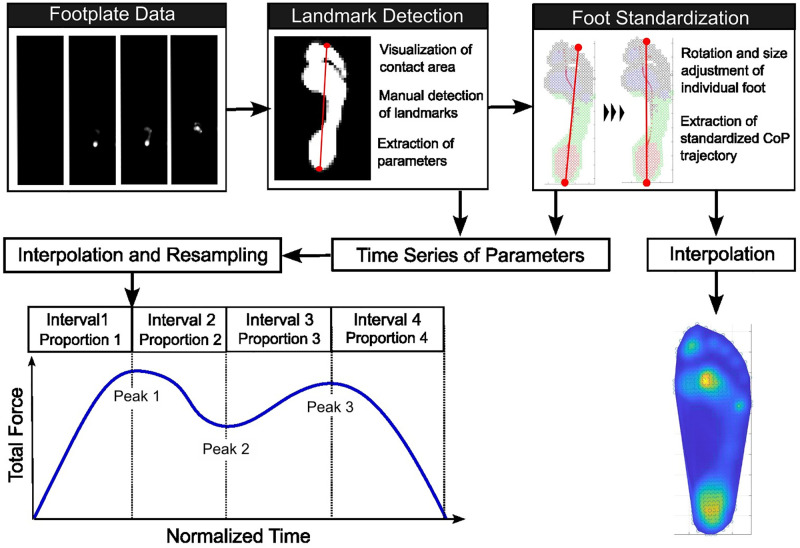
Schematic diagram of the flow of the processing. The top three panels present representative results of (1) the visualization of pressure data (“Footplate Data”), (2) the manual identification of anatomical landmarks (“Landmark Detection”), and (3) the standardization of pressure sensor locations (“Foot Standardization”). The bottom left panel show the characteristic “M-shaped” time-series of the total force, along with definitions of the temporal parameters. The bottom right panel displays an example of the interpolated plantar pressure distribution. See main text for more details.

Foot angle was defined as the angle between the direction of the foot and the direction of progression. In the present study, the direction of the foot was defined as the line connecting two anatomical landmarks: the most posterior point of the heel (Landmark 1) and the tip of the second toe (Landmark 2), as shown by the red line in the “Landmark Detection” panel of Fig 1. These landmarks were visually identified by N.F. and K.F. The direction of progression was defined as the orientation of the long side of the footplate. Positive foot angles were defined in the direction of external rotation. The intra- and inter-rater reliability of manual landmark identification was evaluated. To assess intra-rater reliability, a single rater (N.F.) repeated the identification of Landmark 1 and Landmark 2 ten times using data from the left and right feet of four female and four male participants. The coefficient of variation (CV, %) was calculated for the x- and y-coordinates of both landmarks. The CV ranged from 0.2% to 0.9% for all coordinates, except for the y-coordinate of Landmark 1, for which the CV ranged from 2.8% to 4.8%. For the assessment of inter-rater reliability, the agreement between two raters (N.F. and K.F.) was analyzed for the x- and y-coordinates of Landmark 1 and Landmark 2 using data from the left and right feet of six female and four male participants. The correlation coefficients between the measurements obtained by the two raters were high for all coordinates (*R*² > 0.98).

2.4.2.2 Pressure Parameters. Maximum force, peak pressure and contact area size were computed as the plantar pressure parameters characterizing the plantar pressure distribution pattern during a stance phase. The contact area size (cm²) was computed based on the number of sensors activated during a stance phase. Peak pressure (kPa) was defined as the maximum pressure value recorded by any sensor, and the maximum force (N) was defined as the highest total load recorded during stance phase.

2.4.2.3 Temporal Parameters. The time series of total force during stance phase typically exhibited a triphasic or “M-shaped” pattern [26,27], as shown in the lower left panel of Fig 1. Following heel contact, the load steadily increases, reaching an initial peak (Peak 1) during the load response phase, then decreases to a minimum (Peak 2, often referred to as the “valley” or “trough”) during the mid-stance phase, and finally increases again to hit a second peak (Peak 3) during the pre-swing phase.

To characterize the variation in this temporal structure, contact time was divided into four temporal intervals based on the timings of the three peaks. The durations of these intervals (Intervals 1–4) were computed in milliseconds (ms), along with their relative durations expressed as a percentage of the total contact time (Proportional Intervals 1–4), resulting in a total of eight temporal parameters.

#### 2.4.3. Group comparison of parameters.

Four basic gait parameters, three pressure parameters, and eight temporal parameters were compared between sexes using unpaired *t*-tests with the significance threshold of *p* = 0.05. No correction for the significance threshold was applied due to the exploratory nature of these group comparisons. Foot angle, pressure parameters and temporal parameters were calculated separately for the right and left feet. For these parameters, the average values of the right and left feet were submitted to group comparison.

For the parameters of contact area, maximum force, maximum pressure, and proportional intervals 1–4, we examined whether the significant effect of sex, if any, persisted after controlling for potential confounders using multiple regression analysis. In these analyses, separate linear regression models were fitted for each parameter, with the parameter as the dependent variable and sex, age, BMI, cadence, foot angle, and walking speed as predictors to isolate the effect of sex. Variance inflation factors were < 2.0. Sex was coded as a categorical variable (male = 0, female = 1) in the regression models. BMI was selected as the sole anthropometric variable because it incorporates information on both body weight and height. We focused on proportional intervals 1–4 rather than absolute intervals 1–4 because the temporal structure normalized to actual length of the stance phase was the primary focus of the present study.

#### 2.4.4. Spatial clustering of foot regions with sex difference.

2.4.4.1. Spatial Alignment of Contact Areas. The contact area of each stance phase was spatially aligned across participants by transforming the sensor coordinates according to the procedure schematically illustrated in Fig 1. First, the orientation of each foot was aligned with the line of progression. This was achieved by rotating the foot sensor data around Landmark 1 (the most posterior point of the heel) so that the line connecting Landmark 1 and Landmark 2 (indicated by the red line in the “Landmark Detection” panel of Fig 1) aligned with the direction of the long side of the footplate. Next, foot size was standardized across steps by transforming the sensor coordinates such that the Euclidian distance between Landmark 1 and Landmark 2 was scaled to 100 (A.U.). Thereafter, pressure values in the newly standardized coordinate grid were resampled through interpolation using the MATLAB *meshgrid* function.

2.4.4.2. Detection of Spatial Clusters with Significant Sex Difference. To eliminate the influence of body weight differences [27,37,38], pressure values at each sensor were normalized by dividing the pressure values by body weight prior to further analysis. Subsequently, spatial clusters within the left and right feet were detected exhibiting significant sex differences in plantar pressure at the timing of Peak 1, Peak 2, and Peak 3. For the purpose of ascertaining the robustness of the spatial clustering analysis, the same set of analyses was repeated for the pressure distributions averaged across three neighboring temporal points around Peak 1, Peak 2, and Peak 3.

To evaluate sex differences during the transient periods between neighboring peaks, the same spatial clustering analysis was applied to the plantar pressure distributions at the midpoints between Peak 1 and Peak 2 (Transient Phase 1) and between Peak 2 and Peak 3 (Transient Phase 2).

The plantar pressure distribution at each timing was compared between sexes by unpaired *t*-test in a pixel-by-pixel manner. Spatial clusters were formed by connecting the spatially contiguous pixels with significant sex difference at the significance threshold of *p* = .01. Spatial clusters showing significant sex differences were determined using cluster-based permutation test, employing the cluster-based *t*_*max*_ method to control the family-wise error rate while maintaining statistical power [31]. The empirical distribution of cluster-based *t*_*max*_ was generated by Monte Carlo simulation with 1,000 iterations. Cluster-based significance thresholds was set at *p* = 0.05.

#### 2.4.5. Temporal Clustering of Time Windows with Sex Difference.

The time-series of total force, the averaged pressure, and the contact area size were computed. The total force was defined as the sum of the pressure exerted to all the sensors within the contact area. The averaged pressure was computed by averaging the pressure values of all activated sensors. The total force and averaged pressure at each time-point were weight-normalized by being divided by body weight. Then, the total duration of each time series was normalized to 50 time points by interpolating and resampling the raw time-series data using the spline interpolation method.

Temporal windows with significant sex differences were identified using cluster-based permutation test. The procedure for cluster-based permutation test was essentially the same as described in Section 2.4.4., with the sole exception that temporal clusters, rather than spatial clusters, were formed by contiguous time points showing significant sex differences at the significance threshold of *p* = .01. The method for cluster-based thresholding was identical to that described in Section 2.4.4.2. To assess the robustness of the temporal clustering analysis, we conducted the same analyses using a higher temporal resolution, in which the total duration of each time-series was normalized to 100 time points.

#### 2.4.6. Sex Comparison of COP Trajectories.

The COP trajectory was compared between sexes. The COP at each time point was computed as the pressure-weighted mean of the sensor coordinates. Then, the time-series of the x- and y-coordinates of the COP was computed. The y-axis was defined as the direction parallel to the long side of the footplate, and the x-axis as the direction perpendicular to it. The temporal clusters with significant sex difference were detected in the time-series of COP coordinates along x- and y- axes by the cluster-based permutation tests using the same analytic procedure as that applied to the time-series of pressure metrices described in Section 2.4.5.

## 3. Results

### 3.1. Sex difference in basic gait parameters

The basic gait parameters for male and female participants are summarized in Table 2. Although females tended to walk at a numerically higher speed than males, this difference did not reach statistical significance (*t* = 0.78, *p* = 0.441). Significant sex differences were observed in step duration (*t* = −3.17, *p* = .004), cadence (*t* = 2.89, *p* = .007) and foot angle (*t* = −3.51, *p* = .001): males exhibited longer step durations, lower cadence and greater external foot rotation compared to females.

**Table 2:** Mean and standard deviation of gait parameters. In the parentheses are the standard deviations.

	Female	CI Lower	CI Upper	Male	CI Lower	CI Upper	*t*	*p-value*	Cohen’s *d*
N	68			24					
**Step Length (m)**	0.702	0.688	0.717	0.724	0.697	0.751	−1.46	0.152	−0.36
	(0.06)			(0.063)					
**Step Duration (sec)**	0.49	0.482	0.497	0.524	0.503	0.545	−3.17	0.004**	−0.94
	(0.03)			(0.048)					
**Cadence (steps/min)**	122.99	121.14	124.8	115.60	110.68	120.52	2.89	0.007**	0.84
	(7.56)			(11.41)					
**Speed (m/sec)**	1.44	1.402	1.478	1.4	1.303	1.497	0.78	0.441	0.22
	(0.155)			(0.225)					
**Angle (deg)**	3.04	1.97	4.1	6.35	4.74	7.95	−3.51	0.001**	−0.78
	(4.37)			(3.73)					

**p < .01, uncorrected for multiple comparisons, CI Lower: Lower limit of the 95% confidence interval, CI Upper: Upper limit of the 95% confidence interval.

### 3.2. Sex difference in pressure and temporal parameters

The pressure and temporal parameters for male and female participants are summarized in Table 3. The maximum force exerted on the foot was significantly greater in males than in females (*t* = −8.025, *p* < .001), whereas the maximum pressure did not differ significantly between the two groups (*t* = −1.23, *p* = 0.225). Addi*t*ionally, a significant sex difference was observed in the temporal structure of the total force: Interval 3 and Interval 4 were significantly shorter in females compared to males. In accordance with this, Proportional Interval 3 and 4 were significantly smaller in females than males, whereas the opposite pattern was observed for Proportional Interval 1 and 2.

**Table 3:** Mean and the standard deviations of the pressure and temporal parameters. In the parenthesis are the standard deviations.

	Female	CI Lower	CI Upper	Male	CI Lower	CI Upper	*t*	*p-value*	Cohen’s *d*
N	68			24					
**Interval1 (ms)**	177.7	170.1	185.4	174.8	163.6	186	0.45	0.658	0.1
	(31.3)			(26)					
**Interval2 (ms)**	160.8	155.2	166.3	147.7	135.7	159.7	2.03	0.05	0.53
	(22.7)			(27.8)					
**Interval3 (ms)**	165.4	159.9	170.9	209.6	183.3	235.9	−3.4	0.002**	−1.19
	(22.6)			(61)					
**Interval4 (ms)**	167.3	160.8	173.9	187.9	177.4	198.4	−3.41	0.001**	−0.78
	(26.8)			(24.3)					
**Proportional Interval1 (%)**	26.4	25.6	27.1	24.3	23	25.6	2.77	0.008**	0.65
	(3.1)			(3.1)					
**Proportional Interval2 (%)**	24	23.3	24.7	20.7	18.8	22.6	3.28	0.003**	0.95
	(2.9)			(4.4)					
**Proportional Interval3 (%)**	24.8	23.9	25.7	28.9	25.9	31.8	−2.74	0.011*	−0.87
	(3.6)			(6.9)					
**Proportional Interval4 (%)**	24.9	24.2	25.5	26.1	25.1	27	−2.14	0.038*	−0.46
	(2.7)			(2.2)					
**Contact Area [cm** ^**2**^ **]**	131.2	128.4	134	152.4	147.5	157.3	−7.71	<.001**	−1.83
	(11.5)			(11.3)					
**Max Pressure [kPa]**	550.7	519.1	582.2	585.1	537.3	632.9	−1.23	0.225	−0.27
	(129.3)			(110.8)					
**Max Force [N]**	652.2	630.7	673.7	848.1	802.8	893.4	−8.03	<.001**	−2.08
	(88.3)			(105)					

*p < .05, **p < .01, uncorrected for multiple comparisons, CI Lower: Lower limit of the 95% confidence interval, CI Upper: Upper limit of the 95% confidence interval.

Multiple regression analyses revealed that the observed effects of sex on contact area, maximum force, and proportional intervals 1–4 persisted after controlling for age, BMI, cadence, foot angle, and walking speed (| *t* | > 2.0, *ps* < .04).

### 3.3. Spatial clusters with sex difference in plantar pressure distribution

**Fig 2:**
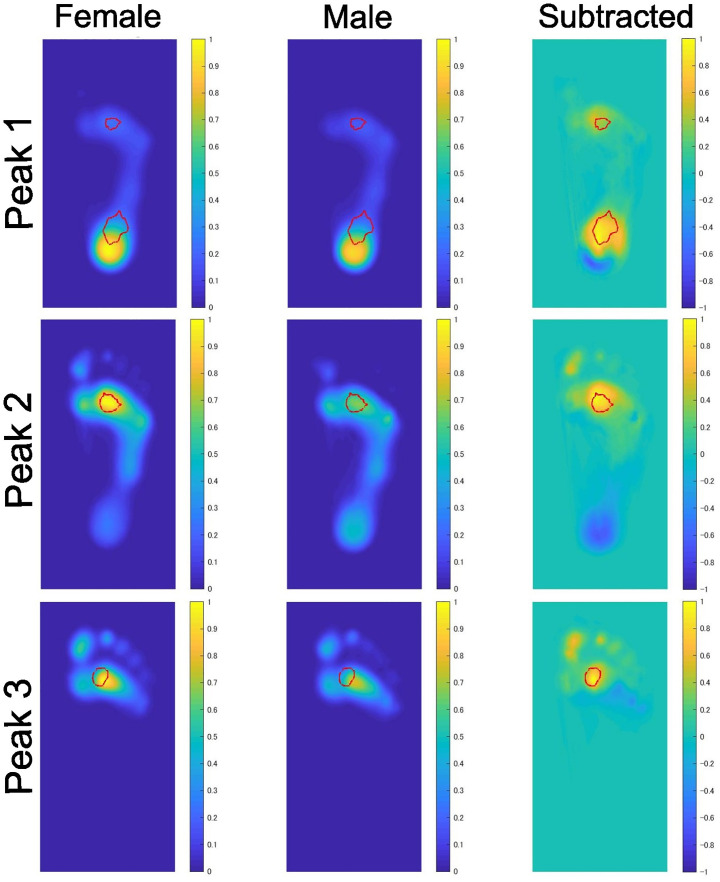
Sex differences in weight-normalized plantar pressure distributions at the time points corresponding to Peak 1, Peak 2, and Peak 3. The leftmost and central panels display the mean weight-normalized pressure distributions for female and male participants, respectively. All pressure values were normalized to [0.0, 1.0], based on the minimum and maximum values observed across both sexes. The rightmost panel presents a difference map, derived by subtracting the male distribution from the female distribution. Spatial clusters showing statistically significant sex differences, as determined by a cluster-based permutation test, are circled in red..

**Fig 3:**
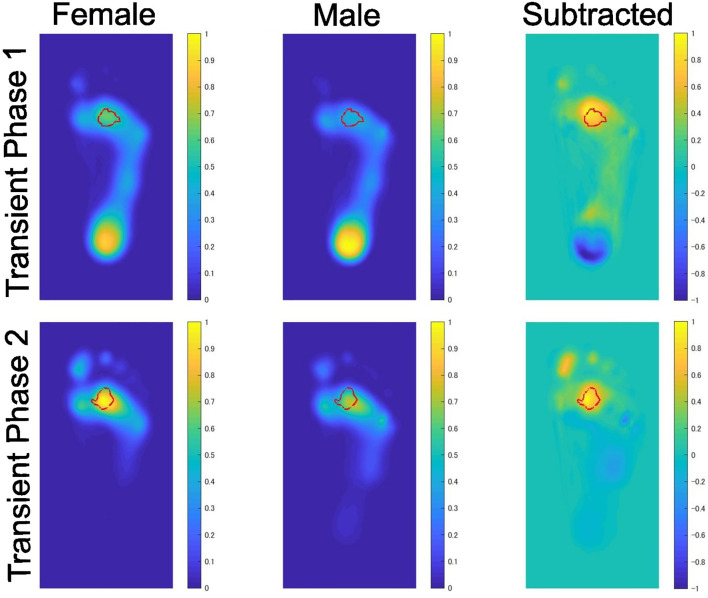
Sex differences in weight-normalized plantar pressure distributions at the time points corresponding to Transient Phase 1 and Transient Phase 2. The data presented are identical to those shown in Fig 2 for these time points..

The averaged distributions of weight-normalized pressure and the results of the cluster permutation test for the left foot are visually presented in Fig 2 and Fig 3. The findings were essentially identical between the right and left feet.

The peak pressure shifted from the calcaneus at Peak 1 to the second metatarsal region at Peaks 2 and 3. At Peak 1, the weight-normalized pressure was significantly greater in females than in males at both the calcaneus and the second metatarsal regions. The sex difference in the calcaneus was no longer statistically significant as early as Transient Phase 1 as shown in Fig 3. At Peaks 2 and 3, regions showing significant sex differences were restricted to the metatarsal region. The results remained essentially unchanged even when averaged pressure distributions across three neighboring time points around Peaks 1–3 were submitted to permutation clustering analysis.

### 3.4. Temporal clusters with sex difference in pressure parameters

**Fig 4:**
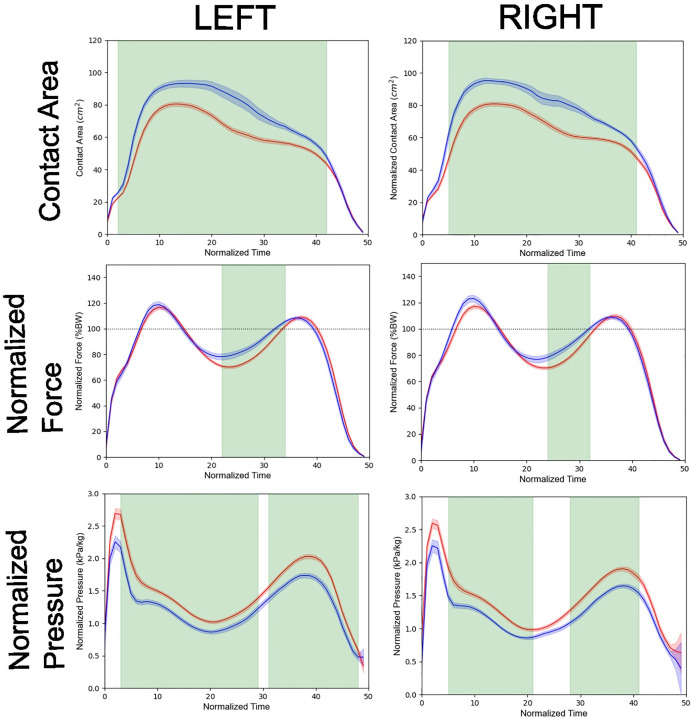
Time series of contact area, normalized total force, and normalized average pressure for the right and left feet in each group. Normalized force was calculated as a percentage of body weight (BW%), using *g* = 9.8m/s^2^. The horizontal dotted line in the panels of normalized force represent 100 BW%. Red and blue lines represent the time series for females and males, respectively. Shaded bands around the lines indicate the standard error. Green-shaded regions represent temporal windows showing significant group differences, as determined by cluster-based permutation test.

Time series of contact area size, weight-normalized total force, and weight-normalized average pressure in male and female participants are shown in Fig 4. As expected, the contact area was larger in males than in females throughout the entire stance phase. The weight-normalized total force was greater in males than in females immediately following Peak 2. In contrast, the weight-normalized average pressure was generally higher in females than in males. Temporal clustering analyses were conducted on time series with higher temporal resolution. The results remained essentially unchanged.

### 3.5. Temporal cluster with sex difference in COP time-series

**Fig 5:**
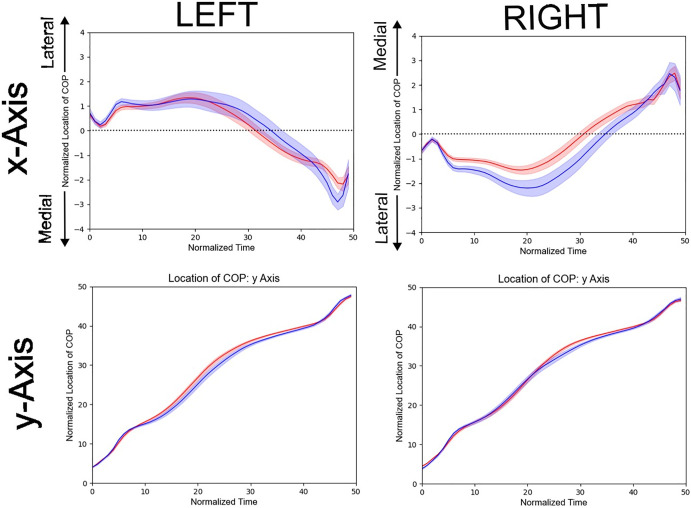
Trajectory of the COP in male and female groups. Red and blue lines represent the normalized COP trajectories for females and males, respectively. Shaded bands around the lines indicate the standard error.

Time series of the COP coordinates along the x- and y-axes in male and female participants are shown in Fig 5. No temporal cluster with significant sex difference was detected. Temporal clustering analyses were conducted on time series with higher temporal resolution. The results remained essentially unchanged.

## 4. Discussion

It has long been recognized that sex differences exist in locomotion patterns [4,5]. Elucidating the specific ways in which locomotor movements differ between sexes is important from both practical and clinical standpoints [6,7]. However, the exiting findings are not necessarily convergent as to the sex difference in the plantar pressure distribution pattern during gait [22–25].

The present study attempted to localize the locus of sex difference in plantar pressure distribution during gait with higher spatial and temporal resolution than the previous studies. To achieve this, we adopted cluster-based permutation statistics, a non-parametric method to detect a spatial or temporal clusters while controlling for the family-wise error rate [30,31]. To the best of our knowledge, the present study is the first to adopt a cluster-permutation approach into the investigation of sex differences in plantar pressure during gait.

The sex comparison of time series data revealed consistently higher weight-normalized plantar pressure in females than in males throughout the stance phase. The results of the cluster-based permutation test localized this sex difference mainly to the calcaneus and the second metatarsal regions.

During the load response phase (Peak 1), weight-normalized pressure was significantly higher in females at both the calcaneus and the second metatarsal regions. The sex difference in the calcaneus was no longer statistically significant as early as Transient Phase 1. During mid-stance (Peak 2) and pre-swing (Peak 3) phases, sex differences in weight-normalized pressure were observed exclusively in the second metatarsal region, with females showing higher pressure values. After the heel contact, the COP moves forward and the body weight is primarily supported by the first and second metatarsal regions and also by the toe near the toe-off. This shift accounts for the localized nature of the sex differences at the later phases.

Previous studies have reported a higher incidence of stress fractures in females compared to males [7]. This phenomenon is often attributed to factors such as osteoporosis [39] and reduced muscle mass [7] in females. However, given that the second metatarsal and calcaneus are among the most common sites of stress fractures [40,41], our findings raise the possibility that biomechanical factors, specifically, repeated exposure to higher plantar pressures in these regions in everyday life, may also contribute to the increased risk in females, consistently with our hypothesis.

Females exhibited a shorter step duration and higher cadence whereas the walking speed and step length were comparable between males and females. This pattern is consonant with the observation that females tend to increase cadence to keep up with certain walking speed [10]. That is, females tend to increase the cadence to compensate for their generally small physique. Zhu et al [42] reported that the increase in cadence heighten the peak pressure across the entire foot regions and that the proportional increase was especially large in the calcaneus [42]. Similarly, in a study that investigated the contribution of cadence to the peak pressure at forefoot revealed that about 20% variance of peak pressure in the second to third metatarsal region is explainable by cadence [43]. Based on these, it is conceivable that the higher cadence explains the higher pressure in females especially in calcaneus and the second metatarsal regions. To address this point, we conducted multiple regression analyses to control for the effects of walking speed and cadence in the analyses of sex differences in pressure and temporal parameters. The significant sex effects on these parameters detected by unpaired *t*-tests persisted after controlling for potential confounders. Unfortunately, a widely accepted method for incorporating covariates into group comparisons using permutation cluster statistics has yet to be developed. Therefore, at this stage, we cannot entirely rule out the possibility that the apparent sex differences observed in the cluster-based permutation analysis are mediated by confounding variables.

Another plausible explanation for the increased pressure observed in the metatarsal region among females is the potential influence of hallux deformities. Hallux valgus, commonly known as a bunion, is a foot deformity characterized by a medial deviation of the first metatarsophalangeal joint, resulting in a prominent bump at the base of the big toe [44]. Relevant to the present study, individuals with hallux valgus typically demonstrate altered plantar pressure distribution [45]. Specifically, there is often a reduction in pressure under the first metatarsophalangeal joint, which is compensated by increased pressure under the second to fifth metatarsophalangeal regions [46,47].

Hallux valgus is reported to be more prevalent among females than males [48,49], which has been attributed to anatomical differences in foot [49] and the more frequent use of constrictive footwear [50] among females. Signs of hallux valgus were not formally assessed in this study, and no participant reported severe foot deformities. However, it is possible that a higher prevalence of mild or undiagnosed hallux valgus among female participants may have contributed to the increased plantar pressure observed in the second metatarsal region.

The analysis of the temporal parameters revealed an unexpected pattern of sex difference. Specifically, the temporal interval of the late stance phase after the Peak 2 (Intervals 3 and 4) was shorter in females than in males, whereas the length of the early stance phase (Interval 1 and 2) was comparable between sexes. This finding contradicts the previous study [51] that increase in walking speed is accompanied by the shortening of all the sub stance phases including load response and mid-stance phase as well as terminal and pre-swing phase. However, their study [51] did not investigate the sex effect. Thus, it is conceivable that it is a sex-specific strategy adopted by females for increasing cadence to shorten only the terminal and pre-swing phase.

There are several limitations to the present study. First, plantar pressure data were collected only during self-selected walking speeds, which severely limited the range of walking speed and cadence in the present study. Consequently, it remains to be seen whether the observed patterns of sex differences would persist under different walking speeds. In addition, both plantar pressure distribution and peak pressure values have been reported to be dependent on cadence [42,43]. Future research is necessary to clarify sex differences in plantar pressure distribution by systematically manipulating gait parameters such as walking speed and cadence.

Second, we cannot exclude the concerns that the observed sex difference derives from the mediation by the confounding variable. Analysis of the anthropometric variables revealed that males had greater body weight, height, and BMI than females. When these variables are included as covariates, the sex-related effects are inevitably reduced in a statistical sense, because there is virtually no overlap in the distributions of these variables between the two sexes in the present dataset. Therefore, to more sensitively evaluate potential sex differences, group comparisons should be conducted between males and females who are carefully matched for anthropometric variables.

As mentioned above, to the best of our knowledge, there is currently no reliable method for controlling covariates in cluster-based permutation statistics. Focusing on weight-normalized pressure distribution and its temporal course likely mitigated the influence of weight differences between the sexes. However, the same set of analyses should be performed in anthropometrically matched groups to test the robustness of the present findings.

Third, foot deformities were not assessed in the participants as stated above. Certain deformities, such as hallux valgus, are known to alter plantar pressure distribution [45] and are more prevalent in females than in males [48]. Formal assessment of the signs of these deformities, even mild ones, must be helpful in elucidating the cause of the observed patterns of sex differences.

Fourth, only one step from each foot was analyzed per participant, which limits our ability to evaluate the influence of step-by-step variability in plantar pressure measurements. It is well known that plantar pressure data obtained using one- or two-step methods exhibit substantial variability [36,52]. Although the midgait method adopted in the present study is regarded as the gold standard and generally provides more stable measurements, recent evidence of significant intra-participant variability [53] suggests that the potential influence of step-by-step variability on our statistical results cannot be completely ruled out.

Fifth, the shape of the plantar surface was not strictly matched across participants, which may have introduced bias into the pixel-by-pixel comparison of plantar pressure distributions between sexes. Previous large-scale studies [54,55] have documented sex differences in foot morphology, particularly in vertical length and circumference. Some of these differences persist even after standardizing foot size by foot length [54,55]. Therefore, it is conceivable that the cluster-permutation test compared pressure patterns between sexes in anatomically non-equivalent regions. Similarly, a closely related study [56] that attempted to match plantar surface shape across participants reported that non-overlapping regions remained even after repeated applications of affine transformations without shearing. To address this limitation inherent in foot shape standardization, more rigorous matching of plantar surface shape should be achieved through the incorporation of anatomical landmarks [57].

## 5. Conclusion

The present study investigated sex differences in plantar pressure distribution during self-paced walking using a novel method of cluster-based permutation statistics. Our findings revealed that females exhibited higher weight-normalized plantar pressures throughout most of the stance phase, particularly in the calcaneus and the second metatarsal regions. These localized pressure differences raises the possibility that biomechanical factors may contribute to the higher incidence of stress fractures observed in females [7].

Additionally, differences in the temporal structure of gait, specifically, shorter terminal and pre-swing phase, indicate sex-specific strategy to increase cadence, hence walking speed, in females. Future research incorporating kinetic and kinematic analyses in combination with plantar pressure evaluation may further elucidate the mechanisms underlying these sex-specific locomotor patterns.
